# More Protein, Less Vitamin D and Omega‐3: An Observational Study of Infant and Toddler Nutrition and Maternal Feeding Attitudes From Türkiye

**DOI:** 10.1002/fsn3.70759

**Published:** 2025-08-04

**Authors:** Beyza Baycan, Yasemin Ertaş Öztürk

**Affiliations:** ^1^ Department of Nutritional Sciences, Institute of Graduate Studies Ondokuz Mayıs University Samsun Türkiye; ^2^ Department of Nutrition and Dietetics, Faculty of Health Sciences Ondokuz Mayıs University Samsun Türkiye

**Keywords:** complementary feeding, dietary intake, infant, mothers attitudes, toddler

## Abstract

This study aimed to evaluate the dietary intakes of infants/toddlers aged 9–24 months and the relationships with their mothers' feeding attitudes. This cross‐sectional study was carried out on 141 mothers with 9–24‐month infants/toddlers. Data were collected through face‐to‐face interviews. The five‐factor Mother's Attitudes towards the Feeding Process Scale (MATFPS) was used to evaluate maternal attitudes. Dietary energy and nutrient intakes were assessed using 24‐h dietary recalls. Weight, length, and neck circumference were evaluated. The mean age of the mothers and infants/toddlers was 31.9 ± 4.59 years and 15.6 ± 5.33 months. The majority of the infants' and toddlers' body weights and lengths were in the normal range with rates of 89.4% and 85.1%, respectively. The distribution of the energy from macronutrients was 41.4 ± 7.51, 44.7 ± 7.02, and 13.7 ± 3.39 for carbohydrates, fats, and proteins, respectively. 98.6%, 90.8%, 46.1%, and 39.7% of infants/toddlers had low dietary intake of EPA + DHA, vitamin D, iron, and fiber, respectively. Energy adequacy of the infants and toddlers was negatively associated with “negative affect during meal” (*r* = −0.175, *p* < 0.05), whereas protein intake adequacy was positively associated with “negative affect during meal” (*r* = 0.250), “attitudes about insufficient/unbalanced feeding” (*r* = 0.201), “reaction to the viewpoints of others” (*r* = 0.178), and the total MATFPS scores (*r* = 0.251, *p* < 0.05). Dietary intakes of infants/toddlers and the attitudes of their mothers are associated. Even though the energy adequacy was high, iron, vitamin D, fiber, EPA, and DHA were below the recommendations in this study. Mothers' attitudes should also be taken into consideration when evaluating the dietary intakes of infants/toddlers. Our findings highlight the importance of supporting parents in similar cultural and nutritional settings through public health strategies that promote adequate dietary intake and appropriate supplement use, for example, iron, vitamin D, and omega‐3 fatty acids.

## Graphical abstract

Dietary intakes of infants/toddlers and the attitudes of their mothers are associated. Even though the energy adequacy was high, iron, vitamin D, fiber, EPA + DHA were below the recommendations. Mothers' attitudes should also be taken into consideration when evaluating the dietary intakes of infants/toddlers. Our findings highlight the importance of supporting parents in similar cultural and nutritional settings through public health strategies that promote adequate dietary intake and appropriate supplement use, for example, iron, vitamin D, and omega‐3 fatty acids.

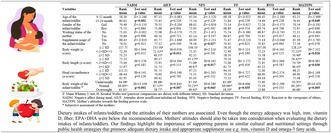

**Abbreviations**
AIUF: attitudes about insufficient/unbalanced feeding
ALA: alpha linolenic acid
BMI: body mass index
DHA: docosahexaenoic acid
EPA: eicosapentaenoic acid
FF: forced feeding
LA: linoleic acid
MATFPS: Mother's Attitudes towards the Feeding Process Scale
NADM: negative affect during meal
NFS: negative feeding strategies
RVO: reaction to the viewpoints of others
SD: standard deviation
TUBER: Nutrition Guideline (Türkiye Beslenme Rehberi)

## 1 Introduction

The complementary feeding phase signifies a pivotal shift in infant nutrition and growth. The transition occurs when exclusive breastfeeding ceases to provide the nutritional requirements of the developing child, generally about 6 months of age (World Health Organization [WHO] 2003). Complementary foods are any food other than breast milk or infant formula that should be offered carefully to improve growth, eliminate nutrient deficiencies, and encourage healthy eating habits (Fewtrell et al. 2017). Long‐term health outcomes including cognitive and immunological development, the avoidance of undernutrition and obesity, and the prevention of chronic diseases depend on optimal nutrition during this age (D'auria et al. 2020; Michaelsen et al. 2017).

The phase of complementary feeding is characterized by the introduction of new foods, flavors, and experiences, as well as major modifications in the diet (Fewtrell et al. 2017). Variations in nutritional consumption may arise not just from the contrast between formula or cow's milk feeding but also from the introduction of diverse quantities and varieties of solid foods (Montalto et al. 1985). Therefore, it is crucial to thoroughly evaluate the consumption of nutrients in infants and closely monitor the intake of essential macro‐ and micronutrients required for their growth and development (Sharma et al. 2013). However, attitudes and practices towards complementary feeding are globally dispersed and often influenced by cultural, regional, and parental factors (Dwijayanti et al. 2024).

Parents have an essential responsibility in the process of introducing solid foods to their infants, which includes determining the timing and content of the food, as well as the method of feeding. This role can influence the interaction between a parent and the infant during the introduction of solid foods, as well as the infant's nutritional status and appetite condition (Fewtrell et al. 2017). As infants are mostly dependent on their mothers during this period and mothers have a significant influence on their infants' nutrition, the feeding attitudes may be a factor affecting infants' food intake. For example, controlled (suppressive or restrictive) feeding practices may disrupt children's self‐regulation of food choice and dietary energy intake early on, which may lead to disturbances in infants' and toddlers' eating behaviors in the future (Faith et al. 2012). In a study including 11–72 month children, maternal attitudes were related to body mass index and having nutritional problems (Bayindir‐Gümüş et al. 2024). Another study showed maternal attitudes, beliefs, and practices had an effect on diet quality and being overweight in 5–9 years old children (Pedroso et al. 2019). Research has demonstrated that maternal eating patterns correlate with maternal attitudes towards the feeding process, and these are linked to feeding difficulties in their preschool children (Tengilimoglu‐Metin and Kabasakal‐Cetin 2024).

Although there is increasing acknowledgment of these processes, the majority of the current study has concentrated on preschool‐aged children, with little focus on the critical period of 9–24 months, which is an exceptionally sensitive stage for the formation of dietary preferences and habits (Werner and Mallan 2024; Maia et al. 2025). Thus, this study aims to fill this gap by assessing both nutrient intake and maternal feeding attitudes in a sample of 9–24‐month‐old children. The aim of the present study was to determine the energy and nutrient intakes of infants and toddlers during the complementary feeding period, the feeding attitudes of their mothers, and show the relationships between them.

## 2 Methods

### 2.1 Participants and Study Design

The study was conducted with 141 mothers and their 9–24‐month infants and toddlers who were registered at the Family Health Centers affiliated with Samsun Public Health Directorate of the Ministry of Health between December 2021 and June 2022. The minimum total sample size was calculated as 111 based on a 0.30 effect size, a 5% margin of error, a 95% confidence interval, and 95% power. The inclusion criteria for mothers were having a 9–24‐month‐old infant or toddler, being between the ages of 19–65, and not having any psychiatric condition. Infants who were premature, had low birth weight, or suffered from chronic or acute diseases at the time of data collection were not included in the study.

Ethics Committee Approval dated 01.10.2021 and numbered 2021/439 was obtained from Ondokuz Mayıs University Clinical Research Ethics Committee, and permission was obtained from Samsun Provincial Health Directorate. Informed consent to participate was obtained from the parents (mothers) of the infant and toddlers before the study data were collected. Data were collected by a questionnaire form consisting of questions about the family, the infant, the infant feeding practices, the Maternal Attitudes towards the Feeding Process Scale, and the food consumption record form.

### 2.2 Anthropometric Measurements

Body weights, lengths, and head circumferences of the infants and toddlers were measured according to the proper technique. Body weight measurements were made on a digital infant scale sensitive to 0.1 kg when the infant or toddler was naked. Length was measured with an infantometer while the infant or toddler was lying down. Head circumference was measured with a rigid tape measure over the ears and eyebrows, as determined by the diameter through the frontal bone and protuberentia occipitalis (Pekcan 2008). Body weight, length, body mass index (BMI), and head circumference were evaluated by calculating the *Z*‐score, according to the WHO standards for 0–5 years, based on the month and gender of the infant. Values between +2 SD and −2 SD were considered normal (WHO 2009).

Body weight and height of the mothers were collected based on their declarations. The BMI of the mothers was calculated using the formula weight (kg)/height (m^2^) and evaluated using the WHO classification for adults as < 18.50 kg/m^2^, underweight; 18.50–24.99 kg/m^2^, normal weight; 25.00–29.99 kg/m^2^, overweight; and ≥ 30.00 kg/m^2^, obese (WHO 2021).

### 2.3 Evaluation of the Mother's Attitudes Towards the Feeding Process

Mothers' attitudes during the feeding process were evaluated using the Mothers Attitudes Towards the Feeding Process Scale (MATFPS), developed by Dilsiz and Dağ (2018) and validated on mothers of children aged 9–72 months. The MATFPS consists of 27 items and five factors (negative mood during meals, attitudes towards inadequate/unbalanced nutrition, negative feeding strategies, forced feeding, and reaction to others' opinions). The scale items are scored on a five‐point Likert‐type scale from “never” to “always.” The total score that can be obtained from the scale varies between 27 and 135 points. An increase in each factor and total scale score indicates an increase in the issues related to the attitudes of mothers towards the feeding process (Dilsiz and Dağ 2018).

### 2.4 Evaluation of the Dietary Intakes

We determined daily energy and nutrient intakes using 24‐h food consumption recall. To improve the precision of dietary evaluation during the complementary feeding period, trained dietitians conducted face‐to‐face interviews using standard portion photo catalog to estimate quantities. Mothers were encouraged to report all foods, beverages, and breastfeeding sessions, including feeding context and frequency. The brand and amount of formula were questioned if the infant consumed formula, and the duration and rate of breastfeeding if they were breastfed. The volume of breast milk consumed during each feeding session was estimated based on duration, assuming an average intake rate of 10 mL/dk. Reported durations clustered around two categories: “5 min or less” and “10 min or more.” Feedings lasting ≤ 5 min were considered as 50 mL, those ≥ 10 min as 100 mL, and intermediate durations were calculated proportionally (e.g., 7 min = 70 mL) according to the previous studies (Aktaç et al. 2015; Emmett et al. 2000).

Nutrition Information System (BeBiS) 7 was used to calculate the daily energy and nutrient intake of infants and toddlers. The energy and nutrient values were compared with the recommendations of the Dietary Guidelines for Türkiye (T.C. Sağlık Bakanlığı 2022). Adequacy below 66% of the recommendations was accepted as “inadequate,” 66%–133% as “adequate,” and above 133% as an “excessive” intake (Yoldaş İlktaç et al. 2021).

### 2.5 Statistical Analysis

We evaluated the data using SPSS version 26.0. Normality was tested with Kolmogorov–Smirnov. Number (*n*) and percentage values were used for categorical data; mean ($\bar{x}$), median (interquartile range) (*M* (IQR)), minimum–maximum, and standard deviation (SD) values were used for continuous data. Independent bivariate data were analyzed using the Mann–Whitney *U* test. Kruskal–Wallis analysis was used for more than two groups followed by Dunn's post hoc tests with Bonferroni correction for pairwise comparisons. The Spearman correlation test was used to assess relationships. A value of *p* < 0.05 was considered significant.

## 3 Results

Table 1 provides descriptive data about the mothers and their infants and toddlers. The mean ages of mothers and their infants or toddlers were 31.9 ± 4.59 years and 15.6 ± 5.33 months, respectively. Almost 50% of the mothers were in the normal BMI classification, 70.9% had a bachelor's degree and/or higher education, and 41.8% were employed. The gender distribution of infants and toddlers was similar, and 73.8% were born by caesarean section. The duration of exclusive breastfeeding was 5.1 ± 1.86 months, and the total duration of breastfeeding was 10.3 ± 6.33 months. On average, infants and toddlers started supplementary feeding at 5.9 ± 0.86 months. Among infants and toddlers, 53.9% used supplements, with vitamin D and iron supplementation usage rates at 52.5% and 30.5%, respectively.

**TABLE 1:** Descriptive data of the mothers and their infants and toddlers.

Variables	Mean ± SD	*M* (IQR)
Mother's age (years)	31.9 ± 4.59	32.0 (7.0)
Infant/toddler's age (months)	15.6 ± 5.33	15.0 (8.0)
Exclusive breastfeeding duration (months)	5.1 ± 1.86	6.0 (1.0)
Total breastfeeding duration (months)	10.3 ± 6.33	10.0 (6.5)
Time to start complementary feeding (months)	5.9 ± 0.86	6.0 (0.2)

Scale scores	Mean ± SD	Min–Max
Negative affect during meal	11.3 ± 6.06	6–30
Attitudes about insufficient/unbalanced feeding	21.7 ± 8.13	8–40
Negative feeding strategies	10.2 ± 4.31	5–22
Forced feeding	4.5 ± 1.65	4–15
Reaction to the viewpoints of others	8.0 ± 4.11	4–20
Total	55.9 ± 19.09	27–119

	*n*	%
BMI classification of the mothers		
Underweight (< 18.5 kg/m^2^)	3	2.1
Normal (18.5–24.9 kg/m^2^)	71	50.4
Overweight (25.0–29.9 kg/m^2^)	47	33.3
Obese (≥ 30 kg/m^2^)	20	14.2
Education of the mothers		
Elementary school	16	11.3
High school	25	17.8
Bachelor's degree and above	100	70.9
Working status of the mother		
Yes	59	41.8
No	82	58.2
Age group of the infant/toddler		
Infant (9–12 months)	61	43.3
Toddler (13–24 months)	80	56.7
Gender of the infant/toddler		
Girl	70	49.6
Boy	71	50.4
Delivery mode		
Vaginal	37	26.2
Caesarean section	104	73.8
Supplement usage of the infant/toddler		
No	65	46.1
Yes	76	53.9
Vitamin D	74	52.5
Iron	43	30.5
Vitamin C	1	0.7
Vitamin B_12_	1	0.7

The *Z*‐score distributions of anthropometric measurements of the infants and toddlers are given in Table 2. Body weight, length, BMI, and head circumference were in the normal range in 89.4%, 85.1%, 77.3%, and 85.1% of the infants and toddlers, respectively.

**TABLE 2:** *Z*‐score distribution of the infants and toddlers.

Anthropometric measurements	*Z* score (SD)					
	≤ −2		< −2 < SD < +2		≥ +2	
	*n*	%	*n*	%	*n*	%
Body weight (g)	2	1.4	126	89.4	13	9.2
Body length (cm)	6	4.3	120	85.1	15	10.6
Body mass index (kg/m^2^)	8	5.7	109	77.3	24	17.0
Head circumference (cm)	2	1.4	120	85.1	19	13.5

Abbreviations: BMI, body mass index; SD, standard deviation.

Table 3 shows the daily energy and macronutrient intakes of infants and toddlers based on the TUBER recommendations. The mean energy intake of the infants and toddlers was 911.6 ± 210.68 kcal, and 78.0% had normal energy intake according to the recommendations. The proportions of energy were 41.4% ± 7.51% from carbohydrates and 44.7% ± 7.02% from fat. The proportions of energy from saturated fat, linoleic acid (LA), and alpha‐linolenic acid (ALA) were 14.4% ± 5.13%, 7.3% ± 3.35%, and 0.5% ± 0.25%, respectively. Protein intake of the infants and toddlers was 2.8 ± 0.92 g/kg, and the majority (97.2%) had high protein intake. Eicosapentaenoic acid (EPA) and docosahexaenoic acid (DHA) intakes were low in 98.6% of the infants and toddlers. The mean adequacy level of fiber intake was 80.9, and 39.7% of infants and toddlers had low intake of fiber. Sucrose intake in the diet was 9.29 ± 7.15 g, and the energy from sucrose was 4.0 ± 3.14 kcal. The adequacy of the iron recommendation level was 79.3%. Iron consumption was identified as insufficient in 46.1% of infants and toddlers. Phosphorus and potassium intakes were high in 95.7% of the infants and 70.2% of the toddlers, respectively. Except for vitamin D, other vitamins were adequate or excessive. The adequacy ratio of vitamin D intake was 26.4; 90.8% of the infants and toddlers had low vitamin D intake from foods.

**TABLE 3:** Evaluation of dietary energy and macro‐ and micronutrient intakes of infants and toddlers according to the recommendations.

Energy and nutrients	Mean ± SD	Recommendation (E%)
Carbohydrate (E%)	41.4 ± 7.51	45–60
Sucrose (E%)	4.0 ± 3.14	As low as possible
Fat (E%)	44.7 ± 7.02	35–40
Saturated fatty acids (E%)	14.4 ± 5.13	As low as possible
Linoleic acid (E%)	7.3 ± 3.35	4
Alpha linolenic asid (E%)	0.5 ± 0.25	0.5
Protein (E%)	13.7 ± 3.39	N/A

	Mean ± SD	%^a^	Adequacy status (*n* (%))		
			Low (≤ 66%)	Normal (67%–133%)	High (> 133%)
Energy (kcal)	911.6 ± 210.68	112.5	4 (2.8)	110 (78.0)	27 (19.1)
Protein (g/kg)	2.8 ± 0.92	256.0	1 (0.7)	3 (2.1)	137 (97.2)
Fiber (g)	8.0 ± 4.37	80.9	56 (39.7)	68 (48.2)	17 (12.1)
EPA + DHA (mg)	74.1 ± 199.89	29.6	139 (98.6)	1 (0.7)	1 (0.7)
Calcium (mg)	559.4 ± 241.36	139.1	10 (7.1)	61 (43.3)	70 (49.6)
Iron (mg)	6.0 ± 3.03	79.3	65 (46.1)	62 (44.0)	14 (9.9)
Zinc (mg)	4.6 ± 1.76	118.8	13 (9.2)	79 (56.0)	49 (34.8)
Magnesium (mg)	125.8 ± 45.40	90.6	41 (29.1)	82 (58.2)	18 (12.8)
Copper (mg)	0.6 ± 0.24	104.1	24 (17.0)	83 (58.9)	34 (24.1)
Sodium (g)	1.1 ± 0.75	163.2	24 (17.0)	44 (31.2)	73 (51.8)
Potassium (mg)	1336.0 ± 505.21	169.2	3 (2.1)	39 (27.7)	99 (70.2)
Phosphorus (mg)	602.6 ± 199.92	267.5	1 (0.7)	5 (3.5)	135 (95.7)
Iyodine (μg)	96.2 ± 36.75	112.4	20 (14.2)	73 (51.8)	48 (34.0)
Vitamin A (μg)	798.1 ± 608.08	329.9	1 (0.7)	11 (7,8)	129 (91.5)
Vitamin C (μg)	67.6 ± 56.79	338.0	6 (4.3)	23 (16.3)	112 (79.4)
Vitamin D (μg)	3.5 ± 3.59	26.4	128 (90.8)	12 (8.5)	1 (0.7)
Vitamin E (mg)	7.7 ± 4.01	134.3	25 (17.7)	52 (36.9)	64 (45.4)
Thiamine (mg)	0.5 ± 0.27	467.3	2 (1.4)	15 (10.6)	124 (87.9)
Riboflavin (mg)	0.9 ± 0.37	177.7	5 (3.5)	33 (23.4)	103 (73.0)
Niacin (mg)	4.4 ± 2.22	280.6	4 (2.8)	13 (9.2)	124 (87.9)
Vitamin B_6_ (mg)	0.8 ± 0.29	183.5	4 (2.8)	39 (27.7)	98 (69.5)
Folate (μg)	127.9 ± 66.84	117.8	28 (19.9)	70 (49.6)	43 (30.5)
Vitamin B_12_ (μg)	2.4 ± 1.53	166.0	21 (14.9)	39 (27.7)	81 (57.4)
Pantothenic acid (mg)	3.2 ± 1.19	88.4	36 (25.5)	94 (66.7)	11 (7.8)
Biotin (μg)	26.9 ± 8.70	195.0	4 (2.8)	41 (29.1)	96 (68.1)

Abbreviations: DHA, docosahexaenoic acid; E, energy; EPA, eicosapentaenoic acid; N/A, not available; SD, standard deviation.

^a^ Adequacy ratio of the recommendation level.

When the consumption status of the main food sources of nutrients shown to be inadequate (EPA + DHA and vitamin D) in infants and toddlers is analyzed, it is found that no participants reported offering fish to their infants or toddlers in the 24‐h recall period. Consequently, the main source of EPA and DHA in the sample was infant formula (*n* = 58, 41.1%). Similarly, the reported use of vitamin D‐fortified foods was very limited.

Table 4 presents the total MATFPS scale and factor scores based on categorical variables. The negative affect during meal factor and total scale score were found to be higher in mothers with toddlers aged 13–24 months. Mothers who did not use nutritional supplements for their infants and toddlers had higher attitudes regarding negative affect during meals compared to those who did. Mothers of infants and toddlers with a body weight *Z*‐score ≤ −2 SD had higher attitudes towards insufficient or unbalanced feeding and total scale scores compared to mothers of infants and toddlers with a body weight *Z*‐score ≥ +2 SD. A statistically significant difference was found among the negative feeding strategies, and total scale scores of among mothers of infants and toddlers with a body length *Z*‐score < −2 < SD < +2 and those ≥ +2 SD. Mothers who characterized their infants and toddlers as underweight compared to their peers had higher scores on all factors of the scale and on the total scale score except on the reaction to the viewpoints of others as given with pairwaise comparisons in Table 4.

**TABLE 4:** Total scale and factor scores according to categorical variables.

Variables	NADM		AIUF		NFS		FF		RVO		MATFPS	
	Rank mean	Test and *p* value	Rank mean	Test and *p* value	Rank mean	Test and *p* value	Rank mean	Test and *p* value	Rank mean	Test and *p* value	Rank mean	Test and *p* value
Age of the infant/toddler												
9–12 month	58.38	*Z* = −3.246 ***p* = 0.001**	67.13	*Z* = −0.983 *p* = 0.326	65.04	*Z* = −1.520 *p* = 0.129	68.58	*Z* = −0.923 *p* = 0.238	66.43	*Z* = −1.180 *p* = 0.238	63.25	*Z* = −1.969 ***p* = 0.049**
13–24 month	80.63		73.95		75.54		72.84		74.49		76.91	
Gender of the infant/toddler												
Girl	70.49	*Z* = −0.150 *p* = 0.880	70.31	*Z* = −0.200 *p* = 0.841	68.05	*Z* = −0.856 *p* = 0.392	73.14	*Z* = −0.927 *p* = 0.354	72.28	*Z* = −0.375 *p* = 0.708	70.34	*Z* = −0.192 *p* = 0.848
Boy	71.51		71.68		73.91		68.89		69.74		71.65	
Working status of the mother												
No	71.01	*Z* = −0.002 *p* = 0.998	72.08	*Z* = −0.370 *p* = 0.711	75.21	*Z* = −1.451 *p* = 0.147	71.74	*Z* = −0.380 *p* = 0.704	68.87	*Z* = −0.743 *p* = 0.457	72.35	*Z* = −0.464 *p* = 0.643
Yes	70.99		69.50		65.14		69.97		73.97		69.12	
Supplement usage of the infant/toddler												
Yes	68.43	*Z* = −0.819 *p* = 0.413	65.77	*Z* = −1.646 *p* = 0.100	64.00	*Z* = −2.211 ***p* = 0.027**	71.47	*Z* = −0.224 *p* = 0.823	71.14	*Z* = −0.046 *p* = 0.963	65.39	*Z* = −1.765 *p* = 0.078
No	74.01		77.12		79.18		70.45		70.83		77.56	
Body weight (*z*‐score)												
≤ −2 SD	117.75	H = 5.944 *p* = 0.051	135.00^a^	H = 9.058 ***p* = 0.011**	106.50	H = 2.150 *p* = 0.341	58.50	H = 3.551 *p* = 0.169	107.00	H = 4.165 *p* = 0.125	128.25^a^	H = 7.332 ***p* = 0.026**
< −2 < SD < +2	72.30		72.32^a,b^		71.35		72.49		72.23		72.21^a,b^	
≥ +2 SD	51.23		48.35^b^		62.15		58.50		53.58		50.46^b^	
Body length (*z*‐score)												
≤ −2 SD	75.33	H = 3.376 *p* = 0.185	60.17	H = 4.394 *p* = 0.111	45.17^a,b^	H = 8.162 ***p* = 0.017**	70.50	H = 1.572 *p* = 0.456	76.50	H = 1.064 *p* = 0.587	58.42^a,b^	H = 6.985 ***p* = 0.030**
< −2 < SD < +2	73.04		73.95		75.08^a^		72.06		71.95		74.70^a^	
≥ +2 SD	52.97		51.73		48.67^b^		62.73		61.23		46.43^b^	
Head circumference (*z*‐score)												
≤ −2 SD	111.25	H = 4.145 *p* = 0.126	91.75	H = 0.546 *p* = 0.761	97.25	H = 1.263 *p* = 0.532	94.50	H = 1.727 *p* = 0.422	72.00	H = 2.374 *p* = 0.305	98.25	H = 1.168 *p* = 0.558
< −2 < SD < +2	68.44		70.90		71.51		70.24		68.90		69.90	
≥ +2 SD	82.95		69.42		65.03		73.32		84.18		75.08	
Body weight of the infant/toddler*												
Underweight	94.98^a^	H = 13.109 ***p* = 0.001**	96.88^a^	H = 9.830 ***p* = 0.007**	87.10^a^	H = 6.280 ***p* = 0.043**	83.48^a^	H = 6.696 ***p* = 0.035**	73.90	H = 2.199 *p* = 0.333	93.95^a^	H = 10.420 ***p* = 0.005**
Normal	69.60^b,c^		67.52^b,c^		70.23^a,b^		69.98^a,b^		72.19		69.26^b,c^	
Overweight	41.45^c^		58.77^c^		49.41^b^		58.50^b^		53.86		46.68^c^	

*Note:* Statistically significant *p* values are shown in bold.

Abbreviations: AIUF, Attitudes about insufficient/unbalanced feeding; FF, Forced feeding; H, Kruskal–Wallis test (pairwise comparisons are shown with different letters); MATFPS, Mother's attitudes towards the feeding process scale; NADM, Negative affect during meal; NFS, Negative feeding strategies; RVO, Reaction to the viewpoints of others; SD, Standard deviation; *Z*, Mann–Whitney *U* test.

* Subjective assessment of the mothers.

The relationships between some quantitative data and the totals as well as factors are given in Table 5. There is a negative correlation between the age of the mothers and both their reaction to the viewpoints of others and the total scale score. A negative correlation was found between the duration of exclusive breastfeeding and the reaction to the viewpoints of others. There is a positive correlation between the total duration of breastfeeding and the mothers' scores on negative affect during meals, and negative relationships with reactions to the viewpoints of others. There is a negative correlation between the energy adequacy ratio and the negative affect during meals. A positive correlation between the energy intake from protein and the negative affect during meals, attitudes about insufficient/unbalanced feeding, as well as the total scale, was found. There is a negative correlation between negative affect during meals and infants' and toddlers' calcium, magnesium, sodium, and vitamin B_6_ adequacy. A negative correlation was observed between negative feeding strategies and calcium, magnesium, and sodium adequacy. There is a negative correlation between forced feeding and infants' and toddlers' sodium and vitamin D adequacy. A positive correlation between the total score and iron adequacy of infants and toddlers was found.

**TABLE 5:** Relationships between quantitative data and total scale and factor scores.

Variables	NADM		AIUF		NFS		FF		RVO		MATFPS	
	*r*	*p*	*r*	*p*	*r*	*p*	*r*	*p*	*r*	*p*	*r*	*p*
Age of the mother (years)	−0.132	0.118	−0.120	0.157	−0.144	0.087	−0.154	0.069	−0.281	**0.001**	−0.209	**0.013**
Exclusive breastfeeding duration (months)	0.006	0.435	−0.028	0.739	0.001	0.995	0.085	0.316	−0.200	**0.018**	−0.029	0.732
Total breastfeeding duration (months)	0.210	**0.013**	0.061	0.476	0.134	0.114	0.144	0.089	−0.041	0.628	0.117	0.167
Energy adequacy (%)	−0.175	**0.038**	−0.080	0.349	−0.124	0.144	−0.099	0.242	−0.034	0.686	−0.121	0.154
Carbohydrate intake (%E)	−0.100	0.239	0.007	0.933	0.025	0.772	−0.061	0.473	−0.013	0.876	−0.023	0.785
Protein intake (%E)	0.197	**0.019**	0.170	**0.043**	0.097	0.255	0.090	0.288	0.160	0.059	0.222	**0.008**
Protein adequacy (%)	0.250	**0.003**	0.201	**0.017**	0.098	0.247	0.088	0.302	0.178	**0.035**	0.251	**0.003**
Fat intake (%E)	0.034	0.686	−0.052	0.541	−0.027	0.750	0.009	0.916	−0.039	0.645	−0.035	0.680
EPA + DHA adequacy (%)	0.117	0.166	0.069	0.417	0.093	0.275	−0.013	0.877	0.107	0.206	0.117	0.169
Calcium adequacy (%)	−0.180	**0.033**	−0.095	0.263	−0.183	**0.030**	−0.128	0.130	0.009	0.914	−0.124	0.142
Iron adequacy (%)	0.152	0.073	0.125	0.140	0.128	0.129	0.028	0.744	0.139	0.099	0.173	**0.040**
Zinc adequacy (%)	−0.048	0.570	0.019	0.822	−0.114	0.179	−0.066	0.434	0.093	0.272	0.005	0.956
Magnesium adequacy (%)	−0.171	**0.043**	−0.040	0.638	−0.197	**0.019**	−0.104	0.221	−0.010	0.904	−0.107	0.208
Sodium adequacy (%)	−0.174	**0.039**	−0.063	0.456	−0.196	**0.020**	−0.208	**0.013**	−0.035	0.676	−0.144	0.089
Potassium adequacy (%)	0.027	0.755	0.008	0.926	−0.065	0.445	0.008	0.925	−0.011	0.895	0.013	0.879
Vitamin A adequacy (%)	−0.026	0.759	−0.009	0.913	−0.050	0.558	−0.116	0.172	0.036	0.674	−0.015	0.858
Vitamin C adequacy (%)	−0.084	0.324	−0.082	0.332	−0.088	0.301	−0.067	0.432	−0.034	0.685	−0.090	0.287
Vitamin D adequacy (%)	−0.129	0.695	−0.011	0.900	0.004	0.964	−0.210	**0.012**	0.060	0.478	−0.033	0.695
Vitamin E adequacy (%)	−0.043	0.612	0.007	0.932	0.075	0.379	−0.015	0.859	0.022	0.793	0.002	0.979
Vitamin B_6_ adequacy (%)	−0.184	**0.029**	0.010	0.906	−0.134	0.113	−0.046	0.588	−0.002	0.978	−0.078	0.360
Folate adequacy (%)	−0.060	0.479	0.037	0.665	−0.082	0.332	−0.119	0.161	−0.038	0.654	−0.018	0.836
Vitamin B_12_ adequacy (%)	0.048	0.570	−0.016	0.853	−0.011	0.895	−0.026	0.762	0.129	0.128	0.055	0.519

*Note:* Statistically significant *p* values are shown in bold.

Abbreviations: AIUF, attitudes about insufficient/unbalanced feeding; DHA, docosahexaenoic acid; E, energy; EPA, eicosapentaenoic acid; FF, forced feeding; MATFPS, Mother's Attitudes Towards the Feeding Process Scale; NADM, negative affect during meal; NFS, negative feeding strategies; RVO, reaction to the viewpoints of others.

## 4 Discussion

The objective of this study was to assess the dietary intakes of infants and toddlers during complementary feeding and examine the attitudes of their mothers towards this process. Additionally, the study aimed to identify any associations between the infants' and toddlers' dietary intakes and their mothers' attitudes.

In the present study, we observed that energy and nutrient intakes were sufficient and, in some cases, even excessive. An imbalance between energy intake and expenditure primarily causes childhood obesity. Underlying disorders or syndromes play a minor role in the development of childhood obesity (Kliegman et al. 2016; WHO 2021). However, in the present study, 89.4% of the infants and toddlers had normal body weight, and 9.2% had excess body weight for age. Given that the energy consumption of the infants in our study was sufficient and not significantly excessive, the body weights of the infants were determined to be within the normal range.

In the present study, the distribution of the energy from macronutrients was 41.4 ± 7.51, 44.7 ± 7.02, and 13.7 ± 3.39 for carbohydrates, fats, and proteins, respectively. The mean energy from sucrose was 4.0% ± 3.14%. Excessive sugar intake starting from a young age has been associated with negative consequences such as obesity, increased risk of cardiovascular disease, and tooth decay (Rupérez et al. 2019). The Turkish Nutrition Guidelines advise minimizing sugar intake throughout early childhood, whereas the ESPGHAN recommends limiting it to < 5% of total caloric consumption (Nataša et al. 2017; T.C. Sağlık Bakanlığı 2022). Although the sucrose intake in the study adhered to ESPGHAN recommendations, it is crucial to further decrease sugar consumption to avoid adverse effects.

The consumption of dietary fats plays a crucial role in promoting brain growth and development throughout the first 2 years of life. It has been emphasized that the quality of fats consumed in early childhood is more important than their quantity (Savarino et al. 2021). ALA and LA are essential long‐chain polyunsaturated fatty acids that play a crucial role in infant nutrition. EPA and DHA synthesized from alpha‐linolenic acid are essential nutrients for growth and normal cellular function. Docosahexaenoic acid is essential for the proper functioning and development of the central nervous system (Gezer and Samur 2012). In one study, EPA and DHA intake among infants and toddlers aged 12–60 months was found to be low at 6 and 20 mg, respectively (Keim and Branum 2015). In the Turkish Dietary Guidelines, it is stated that 100 mg DHA intake is sufficient for 12‐month‐old infants, and 250 mg EPA + DHA intake should be the reference for 24‐month‐old toddlers (T.C. Sağlık Bakanlığı 2022). The evaluation based on TUBER recommendations by age found that 98.6% of infants and toddlers had low EPA + DHA intake. It was also found that the largest amount of EPA and DHA was obtained from infant formulas, and no fish consumption was reported.

To promote optimal growth, it is essential to fulfill the dietary requirements for protein. Inadequate consumption of protein can result in insufficient growth, brain development, and immune system development. Conversely, excessive protein intake could raise the likelihood of infants and toddlers becoming overweight or obese in adulthood, thereby increasing the chance of acquiring chronic health conditions (Savarino et al. 2021). Studies have reported that infants and toddlers have a high protein intake (Ahluwalia et al. 2016; Aktaç et al. 2015; Altınbaş et al. 2020). When the food consumption records of the infants and toddlers in this study were evaluated, it was found that the energy from protein was 13.7% ± 3.39%, and the amount of protein per kg was 2.8 ± 0.92 g/kg. Consistent with prior research, the protein consumption of infants was observed to be high.

Although the benefits of dietary fiber intake in adults include body weight control, reduced risk of cardiovascular disease and type 2 diabetes, and reduced risk of cancer, the health effects of fiber intake in children have been less studied. However, it is known that higher consumption of whole grains is associated with improved diet quality (Finn et al. 2019). In the Turkish Dietary Guidelines, fiber intake for toddlers aged 1–2 years is recommended at 10 g (T.C. Sağlık Bakanlığı 2022). In a study, it was found that fiber intake was 5.2 ± 0.3 g in infants aged 6–11 months and 8.6 ± 0.3 g in toddlers aged 12–23 months, which was below the recommended intake (Ahluwalia et al. 2016). In this study, the dietary fiber intake among infants and toddlers was found to be 8.0 ± 4.37 g, and 39.7% of infants and toddlers had low fiber intake.

In the present study, the intake of many micronutrients was above the recommendations. However, dietary vitamin D intake was inadequate in 90.8% of infants and toddlers. In addition, iron intake is below the recommended level in about half of the infants and toddlers (46.1%). Iron and vitamin D intakes below the recommendations are similar to those found in other studies conducted in İstanbul/Türkiye (Aktaç et al. 2015; Altınbaş et al. 2020). Vitamin D is one of the most important nutrients involved in bone development along with calcium. The vitamin D content of foods and breast milk is quite low. For this reason, in our country, since 2005, the Ministry of Health has been providing 400 IU/day of free vitamin D supplementation to infants aged 0–12 months (T.C. Sağlık Bakanlığı 2022). The relatively low rate of vitamin D intake may be attributed to factors such as inadequate knowledge on the significance of vitamin D intake, supplementation, or availability of fortified foods (Day et al. 2019). Supporting this, in this study, 49.2% of infants and toddlers with low dietary vitamin D intake did not use vitamin D supplements. Iron is one of the most critical micronutrients during the complementary feeding period, and it is often difficult to meet the requirement (D'auria et al. 2020). Iron deficiency anemia is important because it predisposes individuals to infectious diseases, inadequate mental and motor functions, and growth retardation (Bülbül 2004). In studies conducted in different age groups in Türkiye, iron deficiency anemia has been found to have high rates ranging from 30% to 78% (Türk Hematoloji Derneği 2011). In the Turkey Demographic and Health Surveys 2018, the rates of those who consumed iron‐rich foods in the last 24 h were 59.9% in the 9–11‐month period, 69.0% in the 12–17‐month period, and 67.7% in the 18–23‐month period (Hacettepe Üniversitesi Nüfus Etütleri Enstitüsü 2019). It is clear that iron intake during the complementary feeding period is below the recommended level. For this reason, in our country, free iron support is provided to all infants between 4 and 12 months for prevention (T.C. Sağlık Bakanlığı 2022). However, in this study, 56.9% did not use iron supplements in infants and toddlers with low dietary iron intake.

Verbal and non‐verbal relationships between mother and child shape healthy infant feeding. A secure attachment between mother and child does not occur without positive two‐way reciprocation. Infants are likely to have problems in situations such as sleep and feeding if they do not respond to their mothers. In addition, a mother who does not understand her child's hunger or satiety signals and cannot follow her child's emotions may not be able to develop a correct feeding strategy (Yılmazbaş and Gökçay 2013). In the current study, we discovered that mothers with toddlers aged 13–24 months had higher negative affect during meals and higher total scale scores. On the contrary, Aydın and Özaydın (2022) found higher total scale scores in mothers with 9‐ to 12‐month‐old children. We attributed this difference to the higher breastfeeding rate during the 9‐ to 12‐month period in our study. Considering that breastfeeding provides psychological and physiological benefits for the mother and is a source of satisfaction and happiness, it can be assumed that breastfeeding reduces negative emotional states during meals (Topal et al. 2017). However, we also found a positive correlation between mothers' scores on the negative effects of meals and the total duration of breastfeeding. In other words, as the total duration of breastfeeding increased, mothers reported more negative mood problems during meals. In a study, mothers were surveyed about the factors influencing their choice to stop breastfeeding. The top three factors identified were: the child becoming accustomed to eating solid foods (50.0%), the child reaching the age of 2 years (42.9%), and the child frequently feeding during the night or day, leading to fatigue in the mother (39.9%) (Altunel and Özaydın 2022). Considering the items of the negative affect during meals factor in our study (I feel tired/exhausted at the end of meals, my tolerance towards my child decreases during meals, etc.), it was predicted that mothers' problems in this factor increased in parallel with the duration of breastfeeding.

Mothers are known to worry about their infants and toddlers being underweight or overweight. Concern about their infants and toddlers being underweight is associated with eating pressure, and concern about being overweight is associated with restrictive feeding practices (Harrison et al. 2018). This situation emphasizes that mothers should be objective in evaluating the body weight of their infants and toddlers. Sixty‐nine percent of the mothers in the study said their infants and toddlers with a body weight greater than +2 SD were thinner than their peers. Consistent with the findings of Harrison et al. (2018), our study also demonstrated that mothers are often unable to recognize when their infants and toddlers are overweight. This situation is due to mothers worrying more about their babies being underweight than overweight. As seen in this study, these subjective evaluations of body weight affect mothers' attitudes towards the feeding process. Mothers' evaluations of their infants' and toddlers' body weight as underweight showed that these mothers had more problems in their attitudes towards the feeding process compared to when they evaluated their children as overweight. It is seen that mothers who describe their infants and toddlers as underweight, compared to their peers, have more problems with forced feeding than mothers who describe their infants and toddlers as overweight. In addition, mothers of infants and toddlers with a body weight < 2 SD had more problems with attitudes towards inadequate and unbalanced feeding. In our study, it was observed that mothers' assessments of their infants' and toddlers' body weight were not significantly reflected in their infants' energy and macronutrient intake. This aligns with existing literature, which indicates that parental pressure to eat is strongly associated with children's avoidance of eating (Ek et al. 2016). In another study, Yılmaz (2020) found a significant relationship between the child's eating behavior problems and the mean score of the MATFPS.

Mothers of infants and toddlers with normal length had higher scores on the total scale, including negative feeding strategies. In a study, a negative relationship was found between the height percentiles of children aged 2–6 years, the negative mood during meals, and negative feeding strategies (Çavuşoğlu 2022). As height is influenced by various long‐term factors, it is difficult to explain how it is related to mothers' attitudes during the feeding process. Our study found that mothers who did not use nutritional supplements for their infants and toddlers had a higher level of negative affect during meals. In a previous study, a higher total score of MATFPS was found among mothers who requested nutritional supplements to increase their child's appetite or support growth and development. In other words, it is likely that mothers who apply to the outpatient clinic requesting nutritional supplements for their infants develop problematic attitudes in the feeding process (Çavuşoğlu 2022).

Environmental factors can affect both composition of mother's milk and the child's feeding practices. In a study, it was observed that mothers stated that discourses from the environment such as “your milk is low, that's why your baby doesn't want to breastfeed,” “you gave birth to a very weak baby,” and “you can't breastfeed your baby” negatively affected them. According to the same study, a participant mentioned that her mother‐in‐law had a significant role in providing nutrition for her infant initiated complementary feeding after the second month. According to the same study, a participant mentioned that her mother‐in‐law had a significant role in providing sustenance for her infant and even initiated complementary feeding after the second month (Tozluoğlu 2019). Similarly, in our study, we found a relationship between reactions to the viewpoints of others and maternal age and exclusive breastfeeding duration.

It is often unclear whether dietary intakes of children are a cause or a consequence of particular parenting attitudes. In practice, however, both are reported to influence each other (Gubbels et al. 2009). There are limited data in the literature on the relationship between the energy and nutrient intakes of infants and toddlers during the early period of development and maternal attitudes. Studies examining the relationship between parental attitudes and the child's dietary intake have generally been conducted on preschool children (Collins et al. 2014; Pandey et al. 2019). There are studies in which there is no significant relationship between parenting attitudes and children's dietary intake (Gubbels et al. 2011; Pandey et al. 2019), as well as studies in which there is (Kröller and Warschburger 2008). An association between food intake and parental feeding attitudes has been demonstrated, especially for practices such as rewarding, suppressing, and enabling the child to control their food intake. However, this study was conducted with children at risk of obesity (Kröller and Warschburger 2008). In another study, 138 children aged 3–6 years were examined, and no relationship was found between restrictive feeding, pressure to eat, and worrying about the child's excess body weight, and children's energy intake (Pandey et al. 2019). In the present study, we found associations between mothers' negative affect during meals and infants' and toddlers' daily energy adequacy. In addition, the negative affect during meals, attitudes towards insufficient/unbalanced feeding, and reaction to the viewpoints of other factors was associated with infants and toddlers' protein intake. Furthermore, we found relationships between the scale factors and adequacy ratios of some minerals, such as calcium, magnesium, and sodium, and vitamin B6. These data showed that maternal attitudes may shape infants' and toddlers' energy and nutrient intakes.

In this study, it is a strength that we focused on a previously less studied sample regarding the nutritional status of infants and toddlers aged 9–24 months with a robust sample size, using records of their food consumption and maternal feeding practices with a validated tool (MATFPS). Nevertheless, the use of self‐reported 24‐h dietary recall may introduce recall bias. Future studies could benefit from combining self‐reports with objective biomarkers, such as serum vitamin D levels or food diaries, for more precise assessment. Our study is limited by the fact that the quantity of breast milk was each assessed solely by the mothers' self‐reported statements. Future research may also assess the psychometric evaluation of mothers and the behaviors of infants and toddlers, such as appetite. These aspects have the potential to influence maternal attitudes. Although the study sample provides valuable insight into maternal dietary practices in Samsun province, it may not fully reflect the dietary behaviors of mothers in rural areas or other socioeconomically diverse regions. Future studies should consider broader sampling to enhance generalizability. Given the cross‐sectional nature of the study, the observed associations between maternal attitudes and dietary intake should not be interpreted as causal. Longitudinal studies are necessary to investigate possible mediators of this association.

## 5 Concluison

In conclusion, our study showed that maternal feeding attitudes are associated with the energy and nutrient adequacy of infants and toddlers. In addition, mothers tended to perceive their infants and toddlers as thinner than they actually are and experienced attitude problems in the feeding process. Facilitating the feeding practices to enhance convenience and pleasure for both the mother and child will foster a stronger maternal–infant connection and yield beneficial outcomes for the child's nutritional well‐being. For this reason, providing detailed information to mothers about the growth, development, and body weight of their infants and toddlers compared to their peers during routine measurements in family health centers may improve mothers' understanding of their children's health and development.

We assessed that the total duration of breastfeeding was less than the suggested duration. Although complementary feeding is started in this period, it is critical to maintain and encourage breastfeeding throughout infancy. Information about the continuation of breastfeeding can be provided, especially in family health centers.

Infants and toddlers showed low iron and vitamin D adequacy. The Ministry of Health's free iron and vitamin D supplements were not used as expected. We should encourage parents to use these supplements and educate parents on how to increase their consumption of iron‐rich foods. Providing nutrition education to parents on iron‐rich foods, factors affecting iron utilization in the body, iron needs of infants and toddlers, and how to meet these needs may improve the iron intake of infants and toddlers. Additionally, we found that the majority of infants and toddlers had low EPA + DHA intake. Fish and other seafood products should be encouraged in infant diets. When access to fresh fish is limited due to geographic factors, EPA and/or DHA supplementation should be encouraged. Plant‐based omega‐3 sources such as flaxseeds, walnuts, canola oil, and soybean oil could serve as affordable supporting options for low‐income or inland families.

## Author Contributions

**Beyza Baycan:** conceptualization (equal), data curation (lead), formal analysis (lead), investigation (lead), methodology (equal), writing – original draft (lead). **Yasemin Ertaş Öztürk:** conceptualization (equal), methodology (equal), supervision (lead), writing – review and editing (lead).

## Ethics Statement

Ethics Committee Approval dated 01.10.2021 and numbered 2021/439 was obtained from Ondokuz Mayıs University Clinical Research Ethics Committee, and permission was also obtained from Samsun Provincial Health Directorate.

## Consent

The voluntary consent form was signed by the mothers before the study data were collected.

## Conflicts of Interest

The authors declare no conflicts of interest.

## Data Availability

The datasets used and/or analysed during the current study are available from the corresponding author on reasonable request.
